# Caffeoylquinic acid derivatives rich extract from *Gnaphalium pensylvanicum* willd. Ameliorates hyperuricemia and acute gouty arthritis in animal model

**DOI:** 10.1186/s12906-017-1834-9

**Published:** 2017-06-17

**Authors:** Yan Jiang, Yan Lin, Yi-Juan Hu, Xiao-Jun Song, Hong-Hua Pan, Hong-Jian Zhang

**Affiliations:** 1 Dispensary of Traditional Chinese Medicine, Hangzhou First People’s Hospital, 261 Huansha Road, Hangzhou, 310006 China; 2 Department of Pharmacy, Tongde Hospital of Zhejiang Province, 234 Gucui Road, Hangzhou, 310012 China; 3 Key Laboratory of Research and Development of Chinese Medicine of Zhejiang Province, Zhejiang Academy of Traditional Chinese Medicine, 132 Tianmushan Road, Hangzhou, 310007 China

**Keywords:** *Gnaphalium pensylvanicum* Willd., Caffeoylquinic acid derivatives, Hyperuricemia, Acute gouty arthritis, Xanthine oxidase, Pro-inflammatory cytokines

## Abstract

**Background:**

The *Gnaphalium pensylvanicum* willd. is used in China as a folk medicine to treat anti-inflammatory, cough and rheumatism arthritis. The aim of this study was to evaluate the potential of the extract of *G. pensylvanicum* to treat hyperuricemia and acute gouty arthritis in animal model.

**Methods:**

*G. pensylvanicum* extract was evaluated in an experimental model with potassium oxonate (PO) induced hyperuricemia in mice which was used to evaluate anti-hyperuricemia activity and xanthine oxidase (XO) inhibition. Therapies for acute gouty arthritis was also investigated on monosodium urate (MSU) crystal induced paw edema model.

**Results:**

*G. pensylvanicum* extract showed activity in reducing serum uric acid (Sur) through effect renal glucose transporter 9 (GLUT9), organic anion transporter 1 (OAT1) and urate transporter 1 (URAT1) mainly and inhibited XO activity in vivo of mice with PO induced hyperuricemia. The extract of *G. pensylvanicum* also showed significant anti-inflammatory activity and reduced the paw swelling on MSU crystal-induced paw edema model. Meanwhile, 13 caffeoylquinic acid derivatives and 1 flavone were identified by UPLC-ESI-MS/MS as the main active component of *G. pensylvanicum*.

**Conclusions:**

The extract of *G. pensylvanicum* showed significant effect on evaluated models and therefore may be active agents for the treatment of hyperuricemia and acute gouty arthritis.

## Background

Hyperuricemia is characterized by the high level of uric acid (UA) in blood, which may derive from the increase in purine metabolism and impairment of renal excretion of UA. Moreover, hyperuricemia is a key causal factor for the development of gout and many other diseases such as cardiovascular and renal diseases, hypertension and metabolic syndrome, etc. [1]. The terminal product of purine nucleotides degradation is UA. Xanthine oxidase in liver is the key enzyme which catalyzes production of UA, and urate transport proteins in renal are the main transporters for UA clearance [2, 3]. In addition, the crystallization of uric acid crystal within joints and tissues can drive an inflammatory response, therefore, the goal of the clinical treatment of gout is to reduce the Sur level and inflammatory response [4].

Impaired renal excretion of UA rather than UA overproduction was considered to be the major cause of hyperuricemia. Urate transport-related proteins, such as organic anion transporters involved in renal urate excretion and reabsorption, play a key role in maintaining urate homeostasis. Renal tubular handling of UA is dependent on urate transporters such as URAT1, GLUT9 and OAT1. URAT1, which is located in the brush border membrane of the renal tubular epithelial cell is responsible for reabsorption of urate transporter. This protein is encoded by the SLC22A12 gene, which is a genetic risk factor for inducing hyperuricemia [5]. GLUT9 (encoded by the SLC2A9 gene) was found to be a new target for the treatment of hyperuricemia as a high-capacity urate transporter [6]. In addition, the member of organic anion transporter 1 (OAT1, SLC22A6) distributed at the basolateral membrane contribute to renal urate secretion by mediating urate transport across cell membranes in renal proximal tubules [7]. These results suggest that URAT1, OAT1 and GLUT9 might play important roles in regulating urate excretion, and infecting the hyperuricemia. Thus, these urate transporters have proved to be very important targets for hypouricemia drug discovery [5–8].

*Gnaphalium pensylvanicum* willd. is an annual herbaceous plant and widely distributed in many regions of China. It has been used as a folk medicine for the relief of anti-inflammatory, cough and rheumatism arthritis [9]. Our previous study demonstrated that *G. pensylvanicum* extract (GPE) possess many caffeoylquinic acid derivatives and showed potent inhibitory activity against XO in vitro. Meanwhile, caffeoylquinic acid derivatives had been report that they showed potential anti-huperuricemia and anti-gouty arthritis effect [10–13]. In addition, previous pharmacology works on various species of the *Gnaphalium* genus and its components possess XO inhibitory activity, showed significantly anti-hyperuricemia and anti-gouty arthritis [14–16]. Thus, further studies are needed to clarify the exact mechanisms of GPE in improving hyperuricemia and gout. Here, we assessed the effects of GPE with respect to its possible mechanisms by determining the XO, Sur, interleukin-1*β* (IL-1*β*), tumor necrosis factor-*α* (TNF-*α*), and the expression level of mUART1, mOAT1 and mGLUT9 in renal were also carried out to comprehensively study the mechanism of *G. pensylvanicum* against hyperuricemia and gout.

## Methods

### Chemicals and reagents

Potassium oxonate, allopurinol and uric acid were purchased from Sigma-Aldrich Chemical Company. Colchicine tablets (each tablet contains colchicine in a dosage of 0.5 mg) were purchased from Kunming Pharmaceutical Co., Ltd. (Yunnan, China). Commercial kit used for determining uric acid and XO activity were obtained from the Jiancheng Institute of Biotechnology (Nanjing, China). Enzyme-linked immunosorbent assay (ELISA) kits for IL-1*β* and TNF-*α* assay were purchased from Assay R&D Systems. All other reagents used were standard laboratory reagents of analytical grade and were purchased locally. Antibodies against URATl, GLUT9, OAT1 and *β*-actin were purchased from ProteinTech Group (Chicago, IL), Abcam Inc. (Cambridge, MA, USA), and Santa Cruz Biotechnology (USA). Polyvinylidene fluoride (PVDF) membrane was purchased from Merck (Germany). Tissue Protein Extraction Kit was purchased from KeyGEN Biotech. CO., Ltd. (Nanjing, China). Enhanced bicinchoninic acid (BCA) protein assay kit was purchased from Tiangen Biotech (China). Monosodium urate (MSU) crystals were prepared according to the previously described method [17]. Approximately 1 g of uric acid was dissolved and heated in 200 mL of H_2_O with 6 mL NaOH (1 N), adjusted to pH 8.9, cooled overnight at 4 °C, washed, and dried. Needle-like crystals were recovered and were suspended in sterile saline and Twain 80 (20 mg/mL).

### Plant material

*Gnaphalium pensylvanicum* willd. Was collected from Yuhuan by Xi-Biao Zhang, Zhejiang province, PR China in October 2015. It was identified by Licensed Pharmacist Yi-Bo Feng, Tongde Hospital of Zhejiang Province and the voucher specimens (SYSQC20151001) was deposited at the Key Laboratory of Research and Development of Chinese Medicine of Zhejiang Province, Zhejiang Academy of Traditional Chinese Medicine.

### Preparation of plant extracts

The dried *G. pensylvanicum* (500 g) was extracted using 10 L 95% (*v*/v) ethanol for 3 times (3 × 60 min) by heat reflux extraction. Then solvents were removed with vacuum rotary evaporation to yield 34.38 g of residue. The extraction yield of *G. pensylvanicum* was 6.88% (*w*/w) which was kept at −20 °C until used.

### UPLC-ESI-MS/MS analysis of *G. pensylvanicum*

#### Sample preparation

The *G. pensylvanicum* extracts were prepared by ultrasonic extraction of 1.0 g of powdered dried plant material in 20 mL methanol for 1.5 h at room temperature. The extract was filtered and evaporated in a rotary evaporator. The final extracts were dissolved with 2.0 mL and filtered through a 0.25 mm fluorpore membrane (Millipore, MA, USA) prior to injection into the UPLC-ESI-MS/MS system.

### UPLC-ESI-MS/MS analyses

The Acquity Ultra Performance LC-system (Waters, Milford, MA) with TQ detector was employed to analyses. The UPLC analyses were performed using an Acquity UPLC BEH column (2.1 × 100 mm i.d., 1.7 μm) with a binary mobile phase. Solvetn A was 0.1% formic acid aqueous and B was acetonitrile.

The gradient elution at room temperature was as 15–60% (*v*/v) B at 15 min. The flow rate was 0.25 mL/min and the sample volume injected was 1 μL. The detection wavelength was 250 nm. LC-MS analysis was carried out using a Waters TQ Detector equipped with electrospray ionization (ESI) source. The MS was operated in negative mode, and the data were acquired in scan mode using a *m/z* range of 150 to 1000. The data acquisition and analyses was carried out with Masslynx Workstation Software (version 4.1.896).

### Preparation of drugs and test solutions for in vivo methods

All solution and potassium oxonate suspension were prepared dependent on the medium weight of each group. Allopurinol (4 mg/mL), colchicine (100 mg/L), low content *G. pensylvanicum* (LGP) (20 mg/mL), middle content *G. pensylvanicum* (MGP) (40 mg/mL) and high content *G. pensylvanicum* (HGP) (80 mg/mL) were solubilized in 0.5% DMSO and physiological saline. Potassium oxonate was prepared in suspension with 0.9% NaCl solution (30 mg/mL). Monosodium urate crystals were suspended in 0.9% sterile saline (25 mg/mL) and twain 80.

### Experimental animals

60 male Kun-Ming mice (18 ~ 22 g) and 60 male ICR mice (18 ~ 22 g) were obtained from Experimental Animal Center of Zhejiang Academy of Medical sciences. Animals were housed in plastic cages and maintained on a 12/12 h light/dark cycle and had free access to standard pellet diet and tap water. All experimental procedures were approved by the Guidance Suggestions for the Care and Use of Experimental Animals in Zhejiang University.

### Models of hyperuricemia and drug treatment

Uricase inhibitor PO was used to induce the hyperuricemia model in mice, as described previously. Sixty male Kun-Ming mice were divided into six groups: blank, model, allopurinol (40 mg/kg/day), and the GPE (100, 200 and 400 mg/kg/day) groups. The GPE and allopurinol were given continuously every 24 h six days before hyperuricemia was induced. Normal saline was given to the blank group. The dosages of the GPE were based on the effective dosages in the clinic [9]. Mice were injected intraperitoneally with PO (300 mg/kg) to induce hyperuricemia one hour after the last intragastric administration of GPE and allopurinol on the 7th day.

### Models of acute gouty arthritis and drug treatment

The model of acute gouty arthritis was induced by hypodermic injection of 0.1 mL (10 mg) of MSU crystal suspension into the right foot pad. The male ICR mice were divided into 6 groups, and each group including 10 animals. They were blank, model, colchicine (1 mg/kg/day), the GPE (100, 200 and 400 mg/kg/day) groups. Drugs were intragastricly feed once daily for 7 days. On the 7th day, the MSU crystal was injected 1 h later after drugs were given. Drugs continued to be given for 1 day after injection of MSU crystal. Paw swelling was expressed as thickness variation. Paw thickness was measured with digital display micrometer gauge (Shanghai ChuanLu Measuring Tool Co., Ltd) at 0, 1, 4, 12 and 24 h after MSU injection. All doses were expressed as mg per kg body weight of the respective drugs.

### Blood and tissue processing

#### Models of hyperuricemia

On the 7th day, 1 h after final administration, blood samples were collected and allowed to clot for 1 h at room temperature, and centrifuged at 3000 rpm for 10 min to obtain serum. Serum samples were used for the estimation of uric acid level and stored at −20 °C until testing. The liver tissues were also dissected quickly on an ice-plate for the XO assay. The renal were cut into tubes for histological and western blotting analysis. All the samples were frozen at −80 °C until used for assays.

### Models of acute gouty arthritis

Blood samples were collected 24 h after MSU injection and allowed to clot for approximately 1 h at room temperature and then centrifuged to obtain the serum. Serum samples were stored at −20 °C until testing for cytokines levels were assayed. The mice were then sacrificed and paw surrounding tissues were collected for histological analyses and frozen at −80 °C.

### Uricosuric activity in mice

Sur was measured by the enzymatic colorimetric method using a standard diagnostic kit (Jiancheng, Nanjing), according to manufacturer’s instructions.

### Assays inhibition of liver XO activity

The livers were homogenized in ice-cold saline (9 volumes), then centrifuged at 8000 rpm for 10 min at 4 °C. After centrifugation, the lipid layer was carefully removed and the final supernatant fraction was used for XO activity assays. The XO activity in liver was determined using a standard diagnostic kits (Jiancheng, Nanjing). The liver XO enzyme activity is expressed as mol/min per g protein (U/g).

### Western blotting analysis of mGLUT9, mOAT1 and mURAT1 in kidney tissues

Total protein was obtained from the renal using the Tissue Protein Extraction Kit (KeyGEN Biotech. CO., Ltd., China). Briefly, kidney were collected and lysed in appropriate cold lysis buffer contained 1 mM phenylmethylsulfonyl fluoride (PMSF). After centrifugation (15,000 g for 15 min at 4 °C), the total protein was obtained in the supernatant. The protein level was quantified of the BCA protein assay kit (Tiangen Biotech Co., Ltd., China), according to the manufacturer’s instructions. Protein sample (7 mg/mL) was denatured by mixing with an equal volume of 2 sample loading buffer, and boiling at 100 °C for 5 min. Samples were resolved by electrophoresis with 10% SDS-PAGE and transferred onto a PVDF membrane (Merk, Germany). After blocking non-specific binding sites for 3 h with 5% dried skim milk dissolved in PBST (0.05% Tween 20), the membranes were individually incubated for overnight with anti-URAT (1:500 dilution), anti-GLUT9 (1:500 dilution), anti-OAT1 (1:500 dilution) as well as anti-*β*-actin (1:500 dilution). Develop the color of the blot rocking in 3, 3′-diaminobenzidine (DAB) substrate solution. Stop the reaction by pouring out the substrate after the expected band appears, then well rinsed with distilled water repeatedly. Dry the membrane and store it in the dark place. The data were analyzed via densitometry using Molecular Analyst software (Bio-Rad Laboratories, Hercules, CA) and quantitated levels were normalized to their respective blotting from *β*-actin.

### Assessment of paw swelling

The inflammation was quantified by measuring the thickness of the paw at 0, 1, 4, 12 and 24 h after MSU crystal injections.

### Cytokines assay

The levels of IL-1*β* and TNF-*α* in the serum were determined using ELISA kits (Assay, R&D Systems), according to manufacturer’s instructions.

### Histological analyses

#### Models of hyperuricemia

Mice’s kidneys were fixed for 24 h at room temperature in 4% form paraformaldehyde. Paraffin sections (4 μm thick) were prepared from each kidney, stained with hematoxylin and eosin (H & E) and examined under light miscroscope (Eclipse TI-SR, Nikon Instruments Inc., Japan) at a 200× magnification.

### Models of acute gouty arthritis

The paw region of MSU induced gouty arthritis mice was isolated and decalcified in 10% formaldehyde solution, embedded in paraffin and sectioned at 5 μm, followed by staining with H & E and examined under the light microscope at a 200× magnification.

#### Statistical analysis

All results were expressed as the mean ± standard error of the mean (SEM). The one-way analysis of variance (ANOVA) was used to determine the level of significance followed by using GraphPad Prism 5.0 Software (GraphPad Software, San Diego, CA, USA).

## Results

### Screening of chemical compounds in the *G. pensylvanicum*

**Fig. 1:**
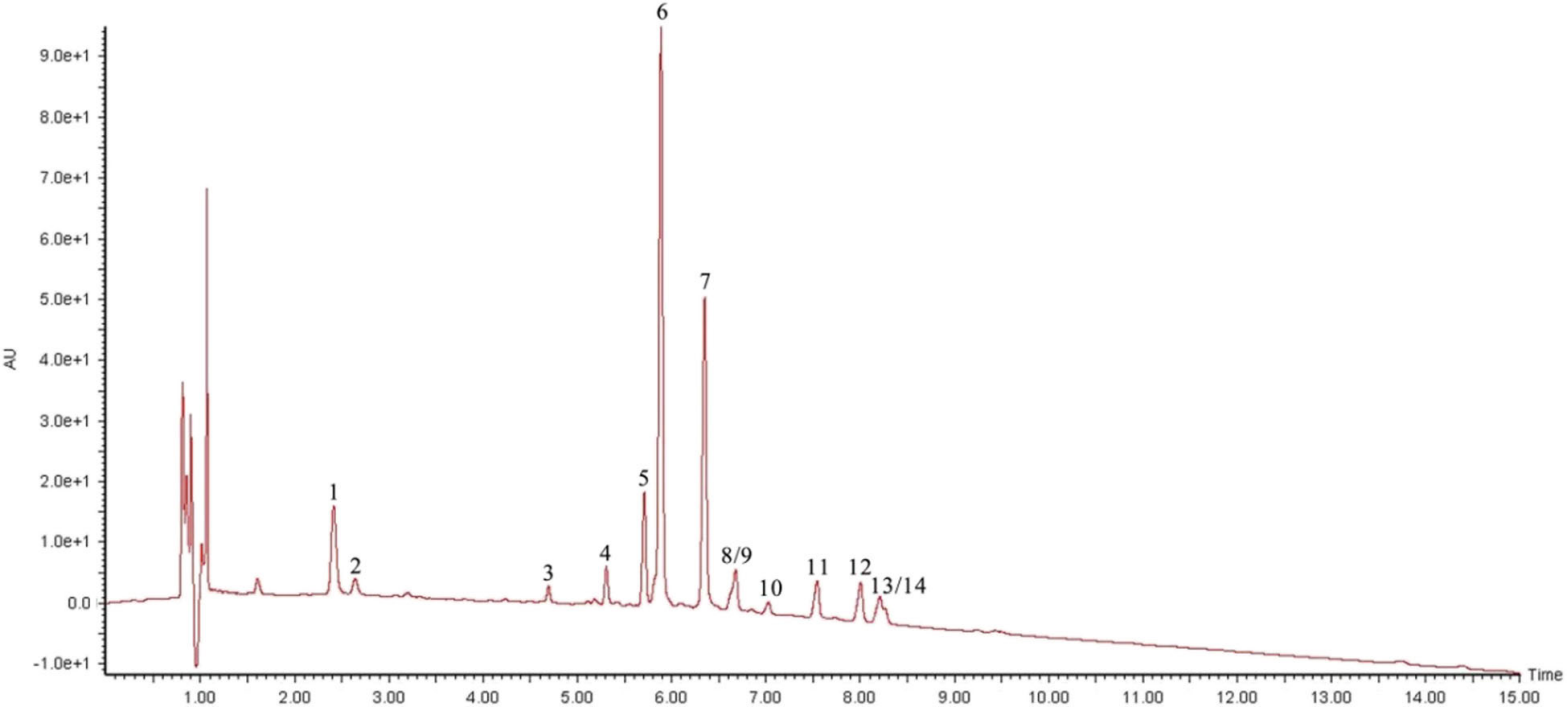
Base peak chromatogram of *G. pensylvanicum* extract by UPLC-ESI-MS/MS in the negative ion mode. Peak labelling represents the compounds identified

The base peak chromatogram that resulted for the GPE is depicted in Fig. 1, where the peaks are numbered according to their elution order. Totally 14 metabolites present in *G. pensylvanicum* were identified by interpretation of their DAD, MS and MS/MS spectra found by UPLC-ESI-MS/MS (Table 1) combined with the data provided in the literature [18–22]. The analysis of the methanol extract revealed that caffeoylquinic acid derivatives were the major classes of secondary metabolites in *G. pensylvanicum*.

**Table 1:** Proposed compounds detected in *G. pensylvanicum* obtained by UPLC-ESI- MS/MS

**Peak**	**Rt (min)**	**UV** **λ**_**max**_ **(nm)**	***m/z***	**MS/MS fragments**	**Proposed compound**	**Reference**
1	2.41	213.7, 325.7	352.9	146.1	3-caffeoylquinic acid	[18–22]
2	2.64	322.7	352.9	249.2	4-caffeoylquinic acid	[18–21]
3	4.69	235.7, 325.7	367.0	308.9, 201.9, 175.3	feruloylquinic acid	[20, 21]
4	5.31	252.7, 345.7	447.1	435.7, 293.5, 106.6	luteolin-7-O-glucoside	[22]
5	5.71	219.7, 242.7, 324.7	515.2	428.8, 334.7, 94.9	1, 3-di-caffeoylquinic acid	[19–21]
6	5.89	218.7, 242.7, 326.7	515.3	490.9, 352.0, 179.1	1, 4-di-caffeoylquinic acid	[19, 20]
7	6.34	218.7, 243.7, 326.7	515.2	352.9, 173.0	3, 4-di-caffeoylquinic acid	[18–21]
8	6.61	244.7, 326.7	515.2	434.1, 346.3, 178.8	4, 5-di-caffeoylquinic acid	[18–21]
9	6.68	243.7, 327.7	601.0	288.4, 240.9	3-methoxyoxaloyl-1, 5-di-caffeoylquinic acid	[22]
10	7.03	243.7, 325.7	528.7	367.4, 172.8, 149.0	1-caffeoyl-3-feruloylquinic acid	[21]
11	7.54	243.7, 326.7	529.2	362.7, 349.2, 179.2	4-caffeoyl-3-feruloylquinic acid	[18, 21]
12	8.01	244.7, 328.7	529.0	374.0, 179.0, 155.6	4-caffeoyl-5-feruloylquinic acid	[18, 21]
13	8.21	243.7, 327.7	557.0	395.4, 233.3, 173.0	acetyldi-caffoylquinic acids	[22]
14	8.26	243.7, 326.7	615.4	453.5, 360.9, 235.8	succinoyl dicaffeoylquinic acids	[20]

### Effects of *G. pensylvanicum* on uric acid levels in serum

**Fig. 2:**
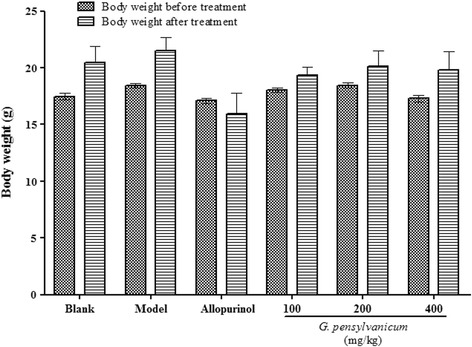
Effects of *G. pensylvanicum* on body weights of hyperuricemia mice. Data are expressed as mean ± SEM (*n* = 10)

As shown in Table 2, compared with blank group (5606.7 ± 1564.9 μmol/L), uric acid level of serum in model group mice (10,675.5 ± 3423.7 μmol/L) was significantly increased after PO administration. Allopurinol, as a positive control drug, significantly reduced uric acid level of serum compared with model group mice (*p* < 0.01). All doses of *G. pensylvanicum* markedly decreased uric acid level a dose-dependent, compared with model group mice. Meanwhile, as shown in Fig. 2, all doses of *G. pensylvanicum* used in this study did not significant effect weight compared with blank group mice. But in the allopurinol group the allopurinol significantly inhibited the weight growth.

**Table 2:** Effects of *G. pensylvanicum* and Allopurinol on Sur and XO active in Liver of PO-Induced Hyperuricemic Mice

**Groups**	**Dose (mg/kg)**	**Sur(**μmol**/L)**	**Liver XO (U/g protein)**
Blank	-	5606.7 ± 1564.9	11.14 ± 1.06
Model	-	10,675.5 ± 3423.7^**^	54.28 ± 8.17^##^
Allopurinol	40	4280.1 ± 2229.5^##^	5.34 ± 0.65^**^
*G. pensylvanicum* extract	100	8388.6 ± 1546.3^**^	10.85 ± 1.53^**^
	200	7288.3 ± 4615.0^**^	8.48 ± 0.85^**^
	400	6012.9 ± 783.6^**^	6.09 ± 0.45^**^

Data represent mean ± SEM for 10 mice; ^#^ *p* < 0.05, ^##^ *p* < 0.01 compared with Blank group (independent samples t-test); ^*^ *p* < 0.05, ^**^ *p* < 0.01 compared with Model group

### Effect of *G. pensylvanicum* on XO activity in liver

As shown in Table 2, PO administration induced a significant advancement of XO activity in liver of model group mice compared with blank group mice (*p* < 0.01). Allopurinol significantly attenuated the XO activity in liver (5.34 ± 0.65 U/g) of hyperuricemia mice model (54.28 ± 8.17 U/g). GPE also significantly exhibited a dose-dependent XO activity in liver of hyperuricemia mice model.

### Effects of *G. pensylvanicum* on expressions of mGLUT9, mURAT1 and mOAT1 in kidney

**Fig. 3:**
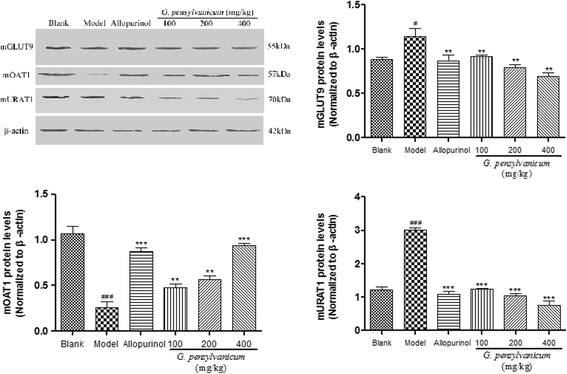
Effects of *G. pensylvanicum* and Allopurinol on protein expression of mGLUT9, mOAT1 and mURAT1 in renal tissues in hyperuricemia mice by Western blot analysis. *N* = 10 per group. ^##^ *p* < 0.01, ^###^ *p* < 0.001 compare with blank; ^**^ *p* < 0.01, ^***^ *p* < 0.001 compare with model

As shown in Fig. 3, compared with blank group, PO administration induced a significant down-regulation of the mOAT1 protein expressions in mice kidney, as well as a significant up-regulation of the mGLUT9 and mURATl protein expression. All doses of *G. pensylvanicum* significantly inhibited the mURATl and mGLUT9 protein expression, and 400 mg/kg *G. pensylvanicum* significantly elevated the mOAT1 protein expressions in hyperuricemia mice kidney, respectively, compared with model group. Allopurinol, could also regulated the expression levels of renal mGLUT9, mOAT1 and mURAT1 protein in hyperuricemia mice compared with model group as showed in Fig. 3.

### Effect of *G. pensylvanicum* on improving renal dysfunction

**Fig. 4:**
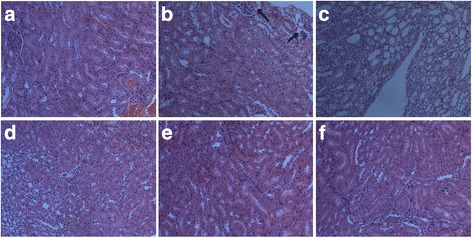
Histology of the kidney, H & E Stain, 200×. (**a**) Blank group, (**b**) Model group with PO, (**c**) Allopurinol 40 mg/kg, (**d**) *G. pensylvanicum* 100 mg/kg, (**e**) *G. pensylvanicum* 200 mg/kg and (**f**) *G. pensylvanicum* 400 mg/kg

As results above, levels of Sur could be restored by all doses of *G. pensylvanicum* in hyperuricemia mice. Our histological data support the observations of uric acid level changes after treatment, and is consistent with the Sur observations in hyperuricemia mice model. Histological examination of renal tissue from the normal mice (Fig. 4a) showed normal renal architecture. Histological analyses showed that the renal tubules were shrank in the model mice (Fig. 4b), compared with the normal mice. These pathological states were ameliorated in some degree by treating with *G. pensylvanicum* (Fig. 4.). However, it is worth noting that allopurinol treatment induced the surface of kidney in allopurinol group showed white were different from the other groups. These results suggested that allopurinol may have the renal toxicity. These results suggested that allopurinol may have the renal toxicity.

### Effects of *G. pensylvanicum* on the MSU crystal-induced mice

**Fig. 5:**
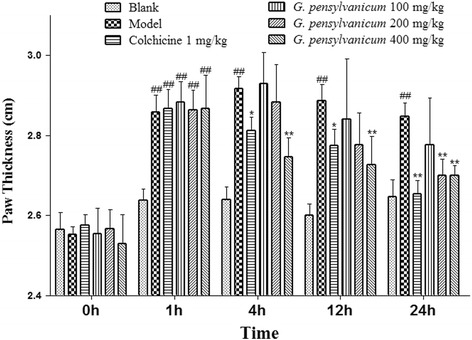
Effect of *G. pensylvanicum* and colchicine on MSU crystal-induced paw edema in mice. Data are expressed as mean ± SEM (*n* = 10). ^##^ *p* < 0.01 compare with Blank group; ^**^ *p* < 0.01, ^*^ *p* < 0.05 compare with Model group

MSU crystals injection caused a significant increase in paw swelling compared with the model group. As shown in Fig. 5, the paw volume of MSU-induced mice revealed an increase in ankle diameter, whereas *G. pensylvanicum* (100, 200, and 400 mg/kg) and colchicine (1 mg/kg) treatment decreases the paw diameter significantly in MSU crystal-induced mice.

**Fig. 6:**
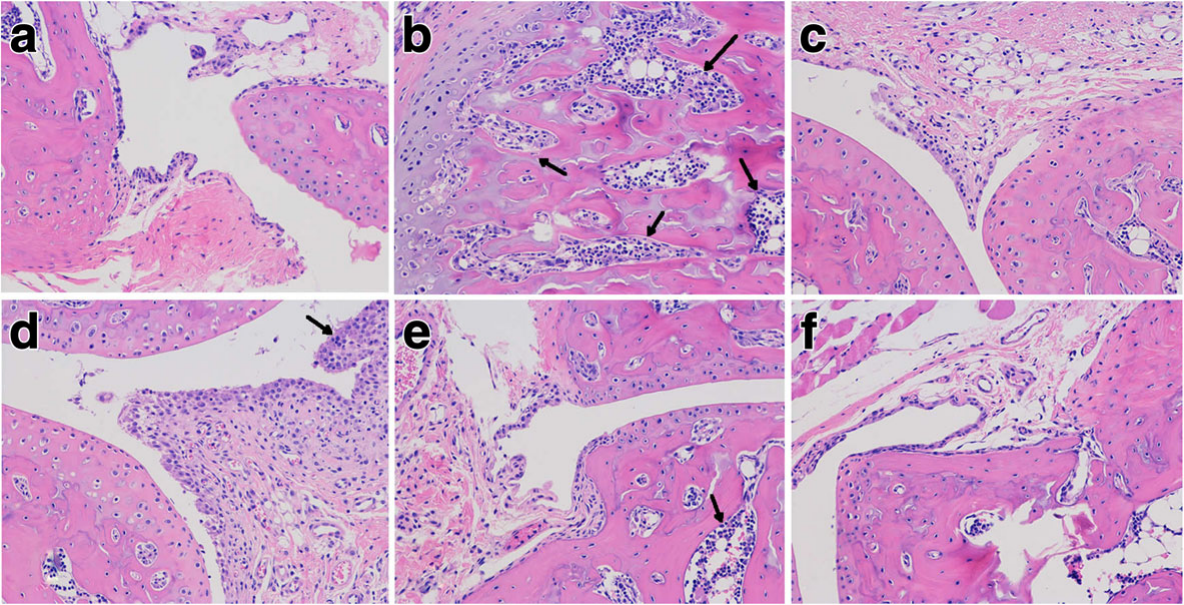
Effects of *G. pensylvanicum* on pathological changes in paw surrounding tissue of mice. All sections were stained with H & E Stain (magnification A-F, ×200). **a** Blank group, (**b**) Model group with MSU, (**c**) Colchicine 1 mg/kg, (**d**) *G. pensylvanicum* 100 mg/kg, (**e**) *G. pensylvanicum* 200 mg/kg and (**f**) *G. pensylvanicum* 400 mg/kg

Joints synovial and surrounding tissues were obtained from the knee joint 2 days after the MSU injection. In the blank control group showed normal and no inflammatory cell infiltration. Model group showed apparent joint inflammation, a number of inflammatory cells infiltration, and hyperplasia synovial. These pathological states were ameliorated in some degree by treating with colchicine and *G. pensylvanicum* dose (Fig. 6). The results demonstrated that *G. pensylvanicum* and colchicine could improve the symptoms of MSU crystal-induced mice, and they could decrease the infiltration of inflammatory cells.

### Effects of *G. pensylvanicum* extracts on the levels of IL-1*β* and TNF-*α* in MSU crystal-induced mice

**Fig. 7:**
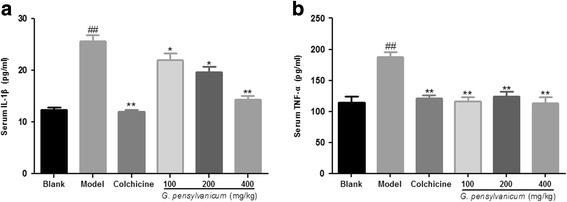
Effects of *G. pensylvanicum* and colchicine on the levels of IL-1*β* (**a**), and TNF-*α* (**b**) in the serum were measured 24 h after hypodermic injection of MSU crystals. Results were shown as mean ± SEM (*n* = 10). ^##^ *p* < 0.01 vs. Blank group; ^*^ *p* < 0.05, ^**^ *p* < 0.01 vs. Model group

To investigate the mechanism of *G. pensylvanicum* in the improvement of paw swelling of MSU crystal-induced mice, the levels of two pro-inflammatory cytokines in the serum were determined. As shown in Fig. 7, MSU crystal induced a significant increase of TNF-*α* and IL-1*β* levels in serum (*p* < 0.01, *p* < 0.01, respectively.). Post-hoc analysis indicated that *G. pensylvanicum* extracts decreased TNF-*α* (100, 200, 400 mg/kg: *p* < 0.01) and IL-1*β* (100 mg/kg: *p* < 0.05; 200 mg/kg: *p* < 0.05; 400 mg/kg: *p* < 0.01, respectively.) levels. In addition, the positive drug colchicine (1 mg/kg) also reduced the TNF-*α* and IL-1*β* levels in serum (*p* < 0.01, *p* < 0.01, respectively.) compared to the model group.

## Discussion

Hyperuricemia is a disorder of uric acid metabolism disease associated with the development of pathological conditions such as gout. Although the disease afflicts humans for years, there were a limited number of drugs currently used in clinical for treating, but there are side effects associated with its use [23, 24]. Natural plant products were the most popular source of complementary and alternative medicines for novel anti-hyperuricemia agents [25].

Gout is a form of inflammatory arthritis characterized by recurrent attacks. And then inflammation is one of the major problems of gout patients has been evaluated [26]. It is well accepted that pro-inflammatory cytokines such as TNF-*α* and IL-1*β* help to propagate the extension of a local or systemic inflammatory process [27]. So, anti-inflammatory was considered as the main method for the treatment of gout.

In this study, *G. pensylvanicum* extracts were able to inhibit the residual activity of xanthine oxidase in vivo and improvement MSU crystal-induced mice paw swelling. Meanwhile, administration of the *G. pensylvanicum* extracts caused a reduction in Sur level in hyperuricemia mice by regulating the abnormal expression levels of mURAT1, mOAT1 and mGLUT9. Thus, *G. pensylvanicum* extracts were able to reduce hyperuricemia and against gouty arthritis through these pathways: increasing uric acid excretion, inhibiting xanthine oxidase activity and pro-inflammatory cytokines secretion, indicating that *G. pensylvanicum* may represent efficacy in the treatment of hyperuricemia and gout. In addition, *G. pensylvanicum* extracts showed renal protection which different from the allopurinol has renal toxicity.

In the phytochemical investigations of *G. pensylvanicum* resulted suggested this plant was rich in caffeoylquinic acid derivatives. Among these compounds, chlorogenic acid [13], 3,5-dicaffeoylquinic acid [11], 4,5-dicaffeoylquinic acid methyl ester [10] and 1,5-dicaffeoylquinic acid [12] showed significant inhibition of xanthine oxidase activity and anti-hyperuricemia. Thus, anti-hyperuricemia effects by uricosuric or inhibition of xanthine oxidase way by the *G. pensylvanicum* extracts can be partially attributed to caffeoylquinic acid derivatives. It could also effectively regulate PO-induced changes of renal mURAT1, mGLUT9 and mOAT1 expressions, resulting in the enhancement of uric acid excretion. However, further investigation is needed to evaluate potential clinical using of *G. pensylvanicum* and its bioactive components. These insights are relevant to further develop the new drugs from *G. pensylvanicum* for the treatment of gouty.

## Conclusions

The present results clearly demonstrated that the *G. pensylvanicum* showed remarkable anti-hyperuricemia and anti-gouty arthritis activity. The tested extract possesses potent uricosuric effect in hyperuricemia mice through effect renal mGLUT9, mOAT1 and mURAT1 mainly and inhibiting XO activity in a certain extent, which are attributable to the enhancement of uric acid excretion and protect hyperuricemia-induced renal dysfunction. This beneficial anti-gouty arthritis effect may be mediated, at least in part, by inhibiting the production of IL-1*β* and TNF-*α*. Meanwhile, the caffeoylquinic acid derivatives were identified by UPLC-ESI-MS/MS as the main active ingredients of *G. pensylvanicum*. Therefore, *G. pensylvanicum* extracts can be considered promising in the treatment of diseases like hyperuricemia and gout. The safety and potential clinical utility of the *G. pensylvanicum* in the uric acid excretion and renal dysfunction reduction needs to be confirmed and evaluate by future studies. In addition, the active contents of *G. pensylvanicum* are engaged to explain.

## Abbreviations

ANOVA: Analysis of variance
BCA: Bicinchoninic acid
ELISA: Enzyme-linked immunosorbent assay
GLUT9: Glucose transporter 9
GPE: *Gnaphalium pensylvanicum* extract
H & E: Hematoxylin and eosin
HGP: High content of *G. pensylvanicum* extract
IL-1*β*: Interleukin-1*β*
LGP: Low content of *G. pensylvanicum* extract
MGP: Middle content of *G. pensylvanicum* extract
MSU: Monosodium urate
OAT1: Organic anion transporter 1
PMSF: Phenylmethylsulfonyl fluoride
PO: Potassium oxonate
PVDF: Polyvinylidene fluoride
SEM: Standard error of the mean
Sur: Serum levels of uric acid
TNF-*α*: Tumor necrosis factor-*α*
UA: Uric acid
URAT1: Urate transporter 1
XO: Xanthine oxidase
